# Distinct uptake, amplification, and release of SARS-CoV-2 by M1 and M2 alveolar macrophages

**DOI:** 10.1038/s41421-021-00258-1

**Published:** 2021-04-13

**Authors:** Jiadi Lv, Zhenfeng Wang, Yajin Qu, Hua Zhu, Qiangqiang Zhu, Wei Tong, Linlin Bao, Qi Lv, Ji Cong, Dan Li, Wei Deng, Pin Yu, Jiangping Song, Wei-Min Tong, Jiangning Liu, Yuying Liu, Chuan Qin, Bo Huang

**Affiliations:** 1 Department of Immunology and National Key Laboratory of Medical Molecular Biology, Institute of Basic Medical Sciences, Chinese Academy of Medical Sciences (CAMS) and Peking Union Medical College, Beijing 100005, China; 2 NHC Key Laboratory of Human Disease Comparative Medicine, Beijing Key Laboratory for Animal Models of Emerging and Remerging Infectious Diseases, Institute of Laboratory Animal Science, CAMS and Comparative Medicine Center, Peking Union Medical College, Beijing 100005, China; 3 State Key Laboratory of Cardiovascular Disease, Fuwai Hospital, National Center for Cardiovascular Diseases, CAMS and Peking Union Medical College, Beijing 100005, China; 4 Department of Pathology, Institute of Basic Medical Sciences, CAMS and Peking Union Medical College, Beijing 100005, China; 5 Clinical Immunology Center, CAMS, Beijing 100005, China; 6 Department of Biochemistry and Molecular Biology, Tongji Medical College, Huazhong University of Science and Technology, Wuhan, Hubei 430030 China

**Keywords:** Innate immunity, Mechanisms of disease

## Abstract

Severe acute respiratory syndrome coronavirus 2 (SARS-CoV-2) invades the alveoli, where abundant alveolar macrophages (AMs) reside. How AMs respond to SARS-CoV-2 invasion remains elusive. Here, we show that classically activated M1 AMs facilitate viral spread; however, alternatively activated M2 AMs limit the spread. M1 AMs utilize cellular softness to efficiently take up SARS-CoV-2. Subsequently, the invaded viruses take over the endo-lysosomal system to escape. M1 AMs have a lower endosomal pH, favoring membrane fusion and allowing the entry of viral RNA from the endosomes into the cytoplasm, where the virus achieves replication and is packaged to be released. In contrast, M2 AMs have a higher endosomal pH but a lower lysosomal pH, thus delivering the virus to lysosomes for degradation. In hACE2 transgenic mouse model, M1 AMs are found to facilitate SARS-CoV-2 infection of the lungs. These findings provide insights into the complex roles of AMs during SARS-CoV-2 infection, along with potential therapeutic targets.

## Introduction

The current outbreak of novel coronavirus disease 2019 (COVID-19) by severe acute respiratory syndrome coronavirus 2 (SARS-CoV-2) has caused considerable harm to human health worldwide^1^. Although the host immune system profoundly influences the pathogenesis of SARS-CoV-2 infection, the detailed immune mechanisms involving the regulation of the viral infection remain largely unclear. Initially, SARS-CoV-2 infection mainly occurs in the lower respiratory tract, especially in the alveoli^2^, based on the patient’s early symptom of a dry cough^3^. The inhalation-generated force may allow SARS-CoV-2 to be passively recruited to the alveoli, where the virus infects angiotensin-converting enzyme 2 (ACE2)-expressing type II pneumocytes, triggering lung damage^4^. Alveoli are air sacs located at the end of bronchioles, where 90%–95% of resident immune cells are macrophages^5,6^. Therefore, before infecting alveolar epithelial cells, SARS-CoV-2 might initially encounter and be taken up by AMs, which are distributed on the alveolar surface and act as the first-line defense by efficiently phagocytosing pathogens or particles^7^. To date, the role of AMs in the pathogenesis of SARS-CoV-2 infection remains uncertain. Some fundamental questions should be addressed, for instance, whether SARS-CoV-2 can replicate in macrophages and whether macrophages are a cellular source to spread SARS-CoV-2.

Macrophages can be polarized to M1 pro-inflammatory phenotype by IFN-γ and lipopolysaccharide or to M2 anti-inflammatory phenotype by IL-4 or other factors^8^. Viral infection may induce M1 polarization of macrophages, which is generally considered to be of paramount importance in viral clearance, due to the release of pro-inflammatory cytokines. These cytokines attract a large number of immune cells such as neutrophils and dendritic cells to the infected site and generate antiviral immunity^9,10^. However, in the case of SARS-CoV-2 infection, macrophages in the lung seem to contribute to an excessive inflammatory response and exacerbate the SARS-CoV-2 infection-caused pathogenesis^11^. Currently, how M1 macrophages directly deal with SARS-CoV-2 remains unclear. M2 macrophages typically phagocytize and degrade debris in lysosomes, thus promoting inflammation resolution and repairing damaged tissues^12,13^. However, the role of M2 macrophages in SARS-CoV-2 infection is elusive too. In this study, we provide evidence that M1 AMs strongly take up, amplify, and release SARS-CoV-2, thus spreading the viral infection, while M2 AMs conversely degrade the virus and limit its spread.

## Results

### M1 rather than M2 AMs efficiently take up SARS-CoV-2

**Fig. 1:**
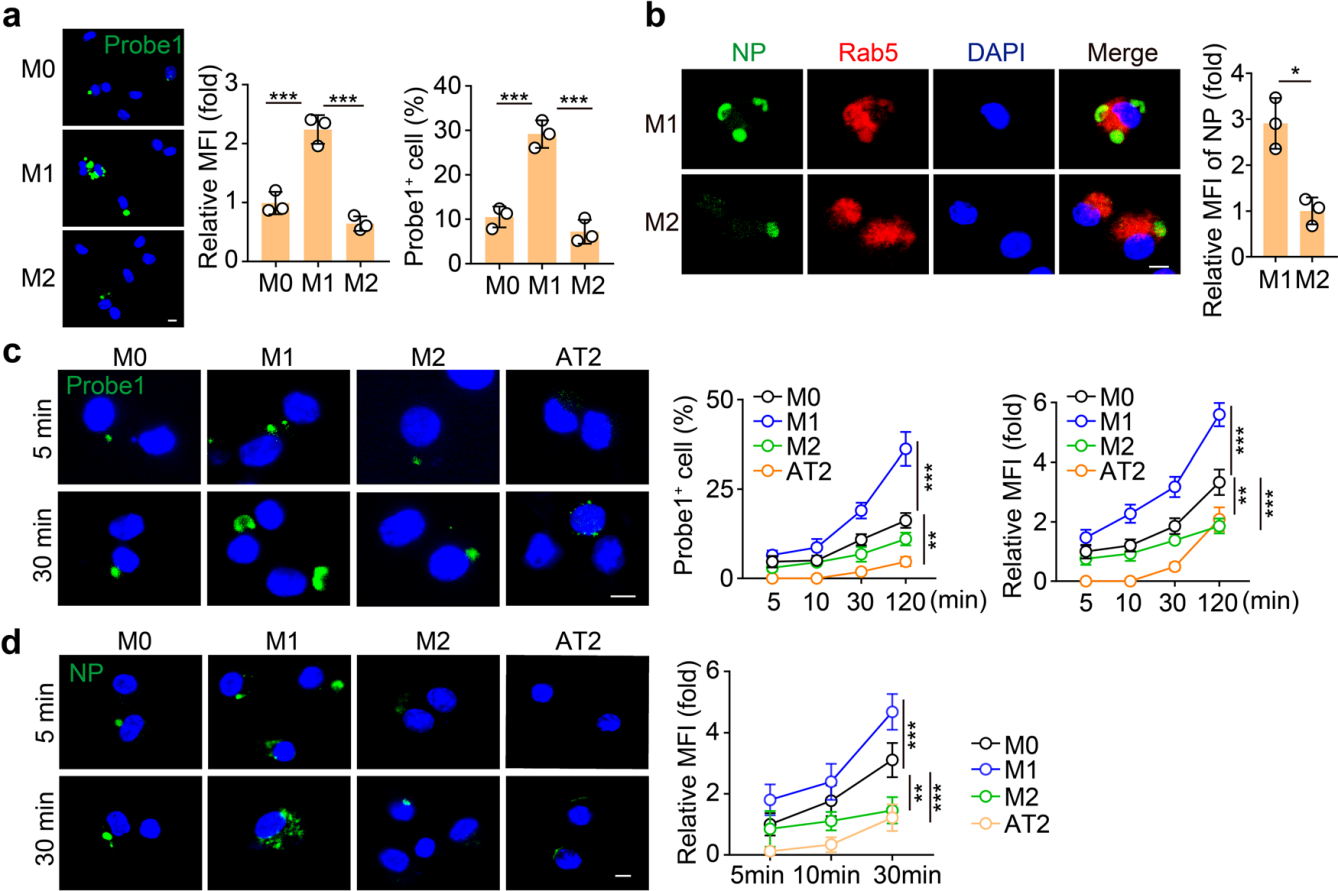
M1 alveolar macrophages can efficiently take up SARS-CoV-2. **a** The primary AMs were polarized to M1 by IFN-γ (20 ng/mL) and LPS (100 ng/mL) or M2 by IL-4 (20 ng/mL) for 24 h, and then infected with SARS-CoV-2 at the ratio of 1:1 (TCID_50_: cells) for 2 h. Cells were fixed for RNAscope analysis with SARS-CoV-2-specific probes. Probe 1 targets viral positive-sense sequence to evaluate viral distribution (green color). Scale bar, 5 μm. **b** M1 or M2 AMs infected with SARS-CoV-2 for 30 min were stained with anti-NP and anti-Rab5 antibodies and observed under a confocal microscope. Scale bar, 5 μm. **c** M0, M1, or M2 AMs and primary alveolar epithelial (AT2) cells isolated from hACE2 mice were infected with SARS-CoV-2 for 5 min or 30 min and then fixed for RNAscope analysis. Scale bar, 5 μm. **d** Same as **c**, except that cells were stained with the anti-NP antibody. Scale bar, 5 μm. The data represent the mean ± SD of 3 independent experiments. *P < 0.05, ***P < 0.001, by one-way ANOVA (**a**, **c**, **d**).

Given that AMs in the alveoli are able to take up SARS-CoV-2, in this study we mainly used primary AMs isolated from murine bronchoalveolar lavage fluid to perform the experiments. The AMs were treated with IFN-γ or IL-4 to generate M1 or M2 AMs, followed by SARS-CoV-2 infection. We found that the viral load was much higher in the M1 AMs compared to the M2 ones (Supplementary Fig. S1a). Performing an RNAscope assay with the green fluorescence-labeled probe against SARS-CoV-2 RNA, we found that substantial green spots were present in M1 AMs only 2 h after infections, and few green spots appeared in M2 AMs at the same time point (Fig. 1a). Similar results were also obtained from mouse Raw264.7 macrophages and the human promonocytic THP-1 cell line (Supplementary Fig. S1b, c), suggesting that M1 rather than M2 macrophages take up SARS-CoV-2. Macrophages take up viruses via phagocytosis, a process using plasma membrane to package viral particles by forming a vesicle called endosome or phagosome in the cytosol^14,15^. Co-staining of Rab5 (an endosome marker) and nucleocapsid protein (NP, a SARS-CoV-2 marker) further showed that M1 AMs phagocytized the virus into endosomes (Fig. 1b). In addition, alveolar epithelial type II (AT2) cells can be invaded by SARS-CoV-2 through an ACE2-mediated pathway^16^. To compare the uptake efficiency between AMs and AT2 cells, we incubated SARS-CoV-2 with AMs (M0, M1, or M2) and AT2 cells for different time lengths. Following a 5 min incubation, around 5% AMs showed the presence of viruses, however, AT2 cells did not show any viral entry, as evidenced by RNAscope (Fig. 1c). This result was supported by the immunostaining of NP, which was observed in AMs but not in AT2 cells (Fig. 1d). However, the viral entry into AT2 cells could be observed after 30 min incubation (Fig. 1c), implying that once SARS-CoV-2 enters the alveoli, AMs may be the first target of the virus. Together, these results suggest that M1 AMs are capable of taking up SARS-CoV-2 with high efficiency.

### SARS-CoV-2 can effectively replicate in M1 AMs

**Fig. 2:**
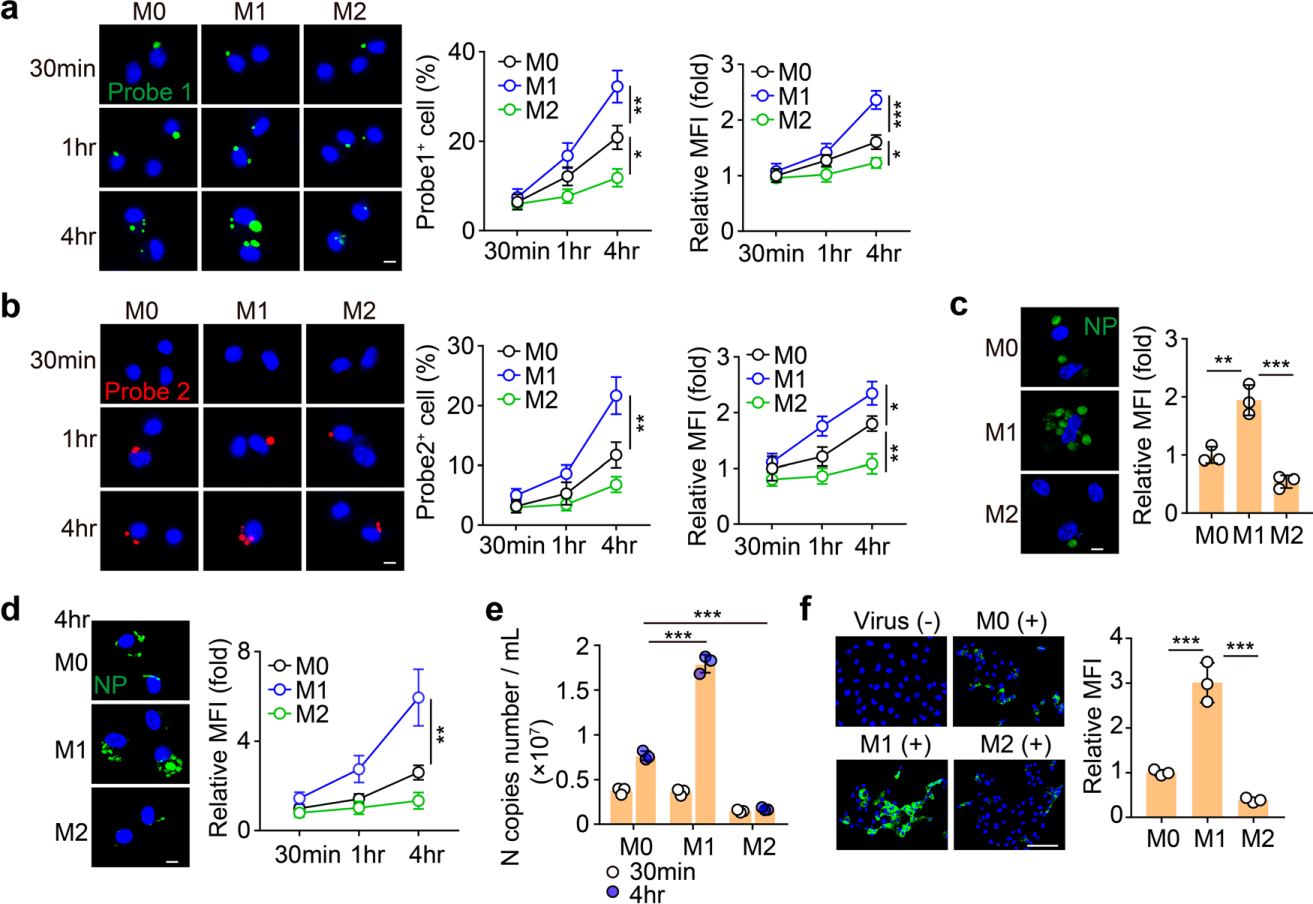
SARS-CoV-2 is apt to replicate in M1 AMs. **a**, **b** M0, M1, and M2 AMs were infected with SARS-CoV-2 for 30 min. Then, the viruses were removed and the cells were cultured with a virus-free medium for another 30 min, 1 h, or 4 h. Cells were fixed for RNAscope analysis. Probe 1 targets viral positive-sense sequence to evaluate viral distribution (green color). Probe 2 targets viral negative-sense sequence to indicate viral replication (red color). Scale bar, 5 μm. **c** Same as **a**, except that cells were cultured with a virus-free medium for another 1 h and stained with the anti-NP antibody. Scale bar, 5 μm. **d, e** Same as **a**, except that cells were stained with anti-NP antibody (**d**) or the viral load from the supernatants was determined by qPCR (**e**). Scale bar, 5 μm. **f** M0, M1, and M2 AMs were infected with SARS-CoV-2 for 30 min. The supernatants were collected to infect Vero E6 cells for 48 h. Cells were stained with anti-NP antibodies and observed by confocal microscopy. Scale bar, 100 μm. The data represent the mean ± SD of 3 independent experiments. *P < 0.05, **P < 0.01, ***P < 0.001, by one-way ANOVA (**a**–**f**).

Next, we investigated whether phagocytized SARS-CoV-2 was able to replicate in the AMs. Following 30 min incubation of SARS-CoV-2 with AMs, we washed and re-cultured the cells in the virus-free medium for an additional 30 min, 1 h, or 4 h. We found that viral loads increased in an exponential fashion in M1 AMs but in a flat fashion in M2 ones, as evidenced by RNAscope and real-time PCR (Fig. 2a and Supplementary Fig. S2a), suggesting that SARS-CoV-2 replicates in AMs. Given that SARS-CoV-2 is a positive-sense single-stranded RNA virus and its replication results in the generation of a negative-sense RNA chain^17^, we also used the red fluorescence-labeled probe to determine the negative-sense RNA by RNAscope. The result indeed showed that following 1 h incubation, red spots were abundant in M1 and rare in M2 AMs (Fig. 2b). In addition, with the prolonged culture time, increased red spots were observed in M1 AMs (Fig. 2b). Together, these results suggest that SARS-CoV-2 can effectively replicate in M1 but not M2 AMs.

### SARS-CoV-2 can be released by AMs

Although the above results indicated that the SARS-CoV-2 genome could replicate in AMs, the cells could possibly block the packaging process, thus generating an intrinsic antiviral response. NP is an important structural protein for SARS-CoV-2 and is critical for the assembly of the nucleocapsid and the release of progeny viral particles^18^. Following the incubation of SARS-CoV-2 with AMs, the immunostaining showed that NP protein was highly expressed in the M1 AMs but barely expressed in the M2 AMs (Fig. 2c). In addition, culturing SARS-CoV-2-treated M1 or M2 AMs with virus-free medium resulted in a strong upregulation of NP protein in M1 but not M2 AMs during the prolonged culture time (Fig. 2d), suggesting that SARS-CoV-2 can achieve the replication and packaging in AMs. Then, we investigated whether the newly synthesized viruses could be released by AMs. Following 30 min incubation of SARS-CoV-2 with AMs (M0, M1, or M2), we washed and re-cultured the cells in the virus-free medium for 30 min or 4 h. We found that a large amount of viral RNA was released to the supernatant by M1 AMs, while much less viral RNA was released by M2 AMs (Fig. 2e). Moreover, the supernatants, especially from the M1 group, were able to infect Vero E6 cells, as evidenced by the immunostaining of NP protein (Fig. 2f). In addition, the macrophages were alive following the incubation with SARS-CoV-2 (Supplementary Fig. S2b), suggesting that macrophages actively release the virus rather than through a passive manner such as cell death. This was validated by using the pan-caspase inhibitor z-VAD-FMK, which prevents cellular death, and did not influence the release of viruses by infected AMs (Supplementary Fig. S2c). Together, these results suggest that SARS-CoV-2 can replicate and be released by AMs.

### Cell softness favors M1 AMs to efficiently take up SARS-CoV-2

**Fig. 3:**
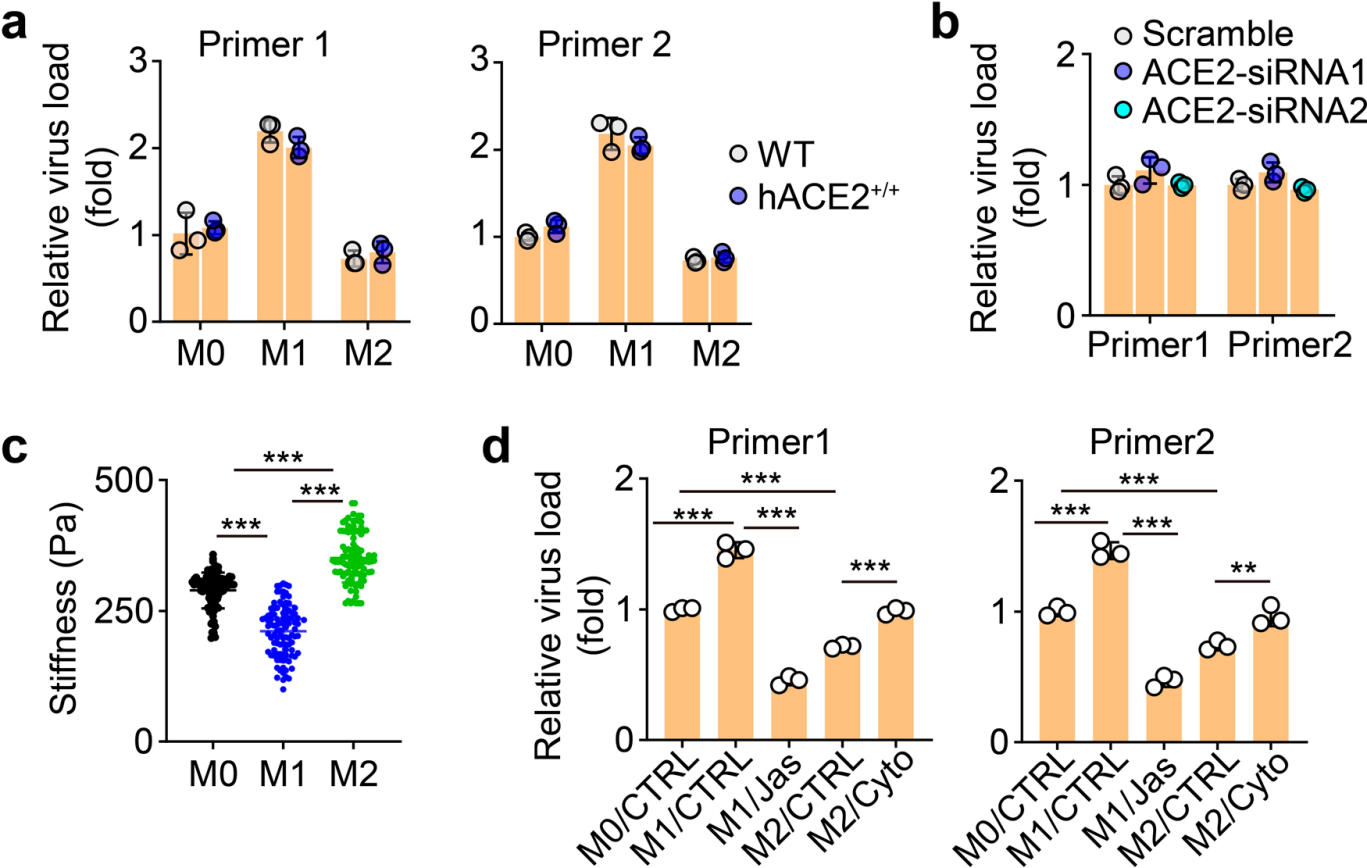
Softness facilitates M1 AMs to take up viruses. **a** AMs isolated from WT or hACE2 mice were infected with SARS-CoV-2 for 2 h. The viral load was analyzed by qPCR. **b** The virus load from *ACE2*-siRNAs AMs was determined by qPCR. **c** The stiffness of M0, M1, and M2 AMs was measured by atomic force microscopy. **d** M1 or M2 AMs were treated with jasplakinolide (Jas, 50 nM) or cytochalasin D (Cyto, 1 μM) for 12 h or 4 h, respectively. Then, AMs were infected with SARS-CoV-2 for 2 h and the viral load was determined by qPCR. The data represent the mean ± SD of 3 independent experiments. **P < 0.01, ***P < 0.001, by one-way ANOVA (**a**, **b** and **d**) or Kruskal–Wallis test (**c**).

The above data indicated that M1 AMs more efficiently took up, amplified, and released SARS-CoV-2 than M2 AMs. Next, we explored the underlying mechanism. ACE2, the receptor that mediates the entry of SARS-CoV-2 into lung epithelial cells, has been reported to be expressed by macrophages^4,19^. We assumed that M1 AMs expressed higher levels of ACE2 than M2 AMs, thus more easily taking up the virus. Unexpectedly, we found that human ACE2-overexpressing AMs did not show enhanced uptake of SARS-CoV-2 (Fig. 3a). On the other hand, knockdown of human ACE2 did not decrease the uptake of the virus by the cells (Fig. 3b and Supplementary Fig. S3a), suggesting that ACE2 is dispensable for M1 AMs to efficiently take up SARS-CoV-2. Cell membrane deformation is required for macrophages to take up extracellular particles. Mechanical softness can be used to reflect deformability. Previously, we have demonstrated that tumorigenic cells use deformability to efficiently take up microparticles^20,21^. We thus measured the stiffness (the inverse of softness) of M1, M0, and M2 AMs by atomic force microscopy. We found that M1 was much softer than M2 AMs (Fig. 3c). It is known that cellular stiffness is mainly mediated by F-actin^22,23^. When we used Cytochalasin D (Cyto), an inhibitor of actin polymerization^24^, to treat M2 AMs, and we found that the cells became soft (Supplementary Fig. S3b), accompanied by an increased ability to take up SARS-CoV-2 (Fig. 3d). A similar result was also obtained with another F-actin inhibitor, Latrunculin A (Lat-A) (Supplementary Fig. S3b, c). On the other hand, increasing M1 AM stiffness using jasplakinolide (Jas), a natural cyclodepsipeptide that is a potent inducer of actin polymerization^24^, led to a decreased uptake of the virus (Fig. 3d). These results suggest that M1 AMs efficiently take up SARS-CoV-2 in a softness-dependent but ACE2-independent way.

### Differential endo-lysosomal pH favors viral replication in M1 AMs

**Fig. 4:**
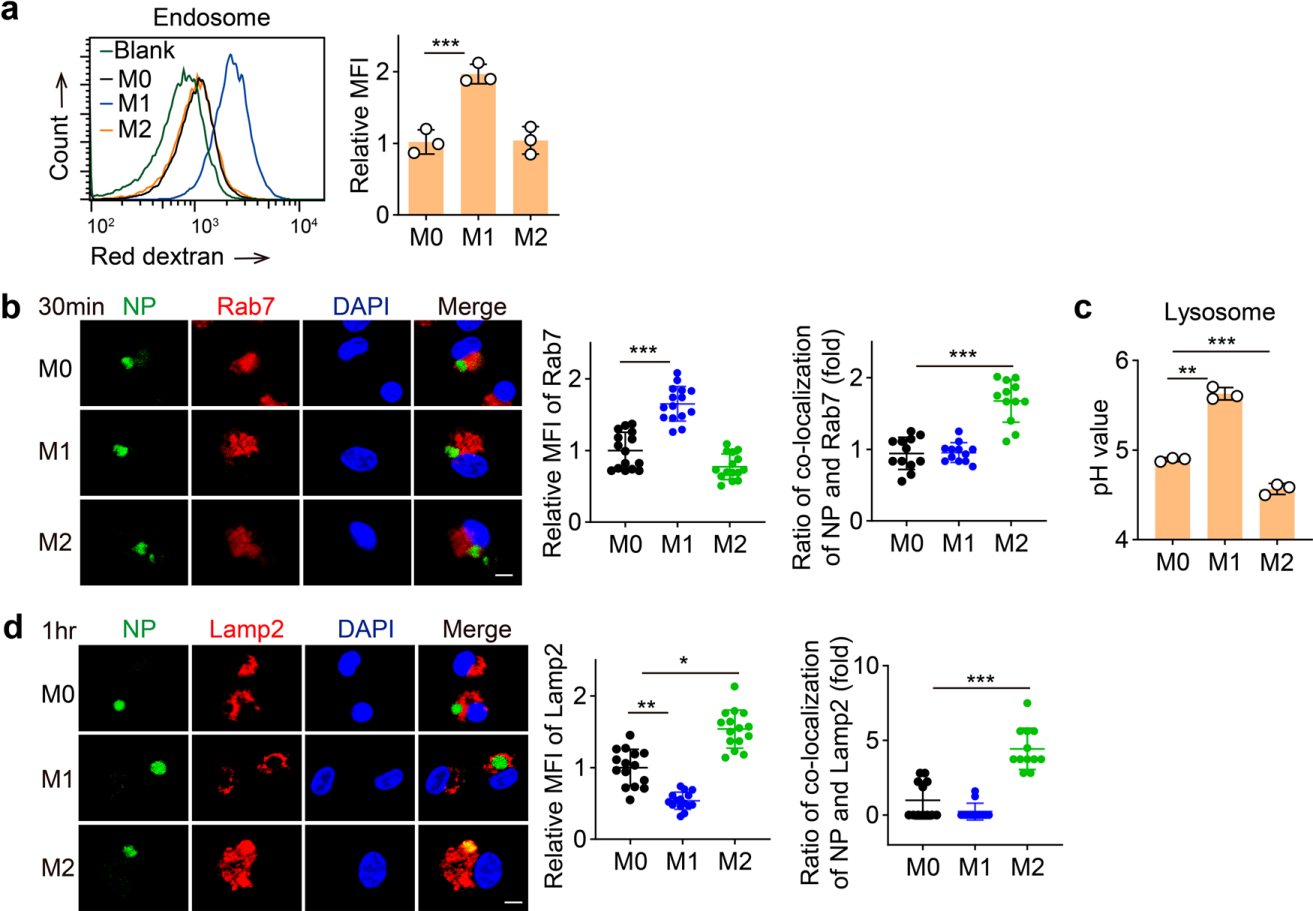
The pH value in endosome or lysosome affects the viral replication in AMs. **a** M0, M1, and M2 AMs were stained with pHrodo™ Red dextran and the fluorescence intensity was determined by flow cytometry. **b, d** AMs were infected with SARS-CoV-2 for 30 min (**b**) or 1 h (**d**), and then stained with anti-NP, anti-Rab7, and anti-lamp2 antibodies. Scale bar, 5 μm. **c** M0, M1, and M2 AMs were stained with LysoSensor™ Yellow/Blue DND-160 and the pH value was detected by a microplate reader. The data represent the mean ± SD of 3 independent experiments. *P < 0.05, **P < 0.01, ***P < 0.001, by one-way ANOVA (**a**–**d**).

Following cellular uptake, the virus enters the endo-lysosomal degradation system of the macrophages. We thus investigated how SARS-CoV-2 escapes this degradation process. Studies have demonstrated that endosomal acidification results in the cleavage of the viral spike protein, leading to the fusion of the viral envelope with the endocytic membrane and allowing the release of the viral RNA genome into the cytoplasm to initiate viral protein synthesis and RNA replication^25^. We used acid-sensitive dextran to measure the endosomal pH since low endosomal pH causes dextran to generate more red fluorescence. Flow cytometric analysis showed a lower endosomal pH in M1 AMs, compared to M2 ones (Fig. 4a). A consistent result was obtained by fluorescent microscopy (Supplementary Fig. S4a). To further validate this result, we incubated SARS-CoV-2 with AMs for 1 h and co-stained the cells with anti-viral NP and anti-Rab7, a marker representing late endosomes that are more acidic than early endosomes^26^. We found that M1 AMs highly expressed Rab7 and that Rab7 was rarely colocalized with NP (Fig. 4b). In contrast, M2 AMs showed colocalization of NP with Rab7 (Fig. 4b). Late endosomes are ready to fuse with Golgi-derived and hydrolytic enzyme-containing vesicles to form lysosomes, where their cargo is degraded by acid-dependent enzymes^27,28^. Intriguingly, compared to M1 AMs, lysosomes were more abundant in M2 AMs, where the colocalization with NP could be observed (Fig. 4d). Moreover, a high lysosomal pH (5.63) and a low lysosomal pH (4.56) were found in M1 and M2 AMs, respectively (Fig. 4c and Supplementary Fig. S4b). Such an acidic pH is likely to facilitate M2 AMs to degrade the viruses in their lysosomes. Together, these results suggest that M1 AMs favor the escape of SARS-CoV-2 genomic RNA from more acidic endosomes but M2 AMs mobilize more acidic lysosomes to degrade the virus.

### Chloroquine (CQ) enhances endosomal acidification in AMs

**Fig. 5:**
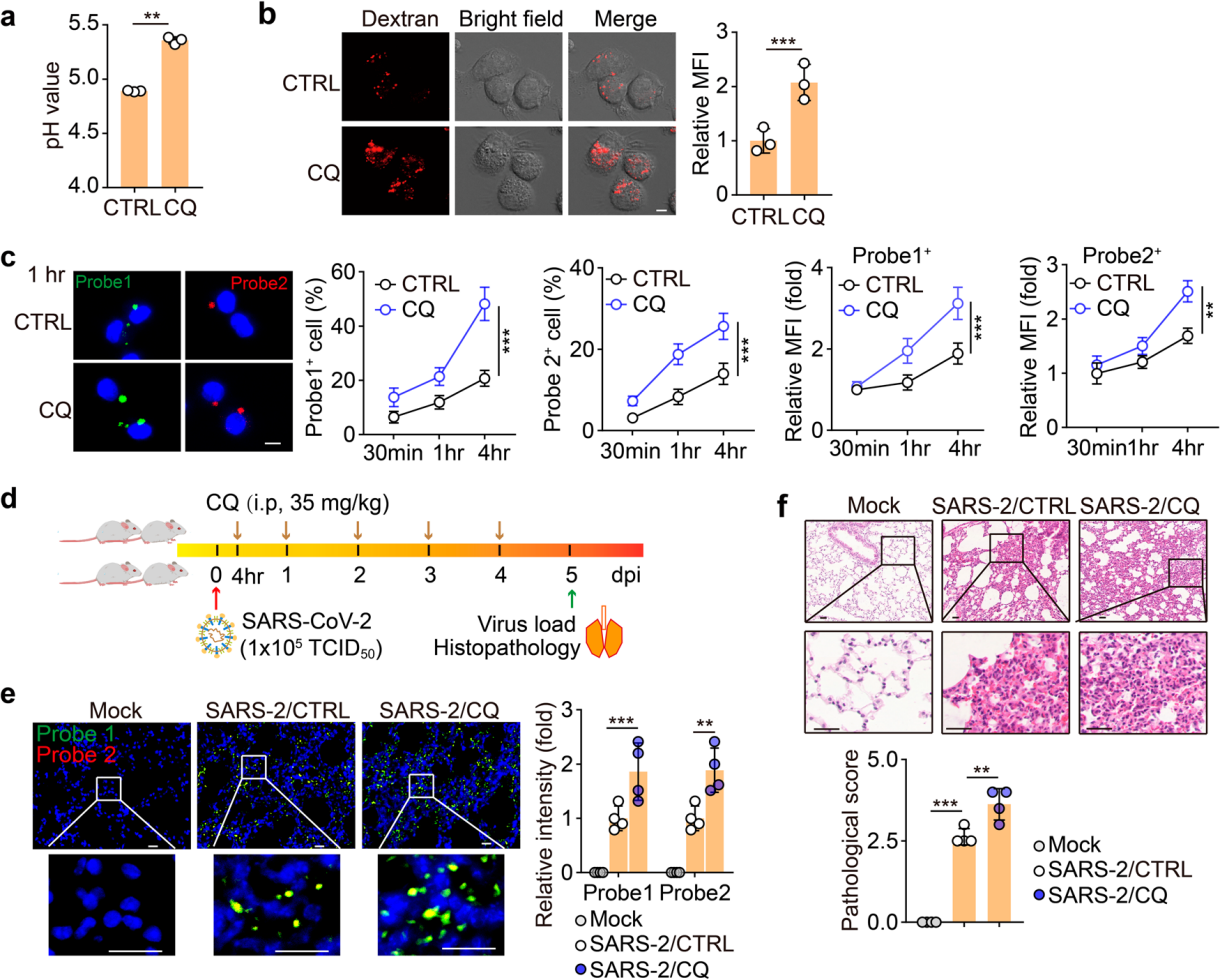
CQ enhances SARS-CoV-2 infection by polarizing M0 macrophages to M1 ones. **a** AMs pretreated with CQ (10 μM) for 24 h were stained with LysoSensor™ Yellow/Blue DND-160 and the pH value was detected by a microplate reader. **b** AMs pretreated with CQ (10 μM) for 24 h were stained with pHrodo™ Red dextran and observed by confocal microscopy. Scale bar, 5 μm. **c** AMs pretreated with CQ (10 μM) for 24 h were infected with SARS-CoV-2 for 30 min, and then cells were cultured with a virus-free medium for another 30 min, 1 h, or 4 h. Cells were fixed for RNAscope with Probe 1 (green color) and 2 (red color). Scale bar, 5 μm. **d**–**f** The schematic diagram of experimental design (**d**). hACE2 transgenic mice were infected with SARS-CoV-2 and then treated with CQ (i.p., 35 mg/kg) once every day for 5 days. The control group (CTRL) received vehicle (water) as a placebo. The lung tissues were fixed to perform the RNAscope analysis with probes 1 (green color) and 2 (red color) (**e**, *n* = 4 mice) and H&E staining (**f**, *n* = 4 mice). Three lung sections from the left lobe were evaluated for each mouse. The representative image reflected the distributions of damaged lung tissues. Scale bar, 20 μm for **e**, 50 μm for **f**. The data represent mean ± SD. In **a**–**c**, *n* = 3 independent experiments. **P < 0.01, ***P < 0.001, by two-tailed Student’s *t-*test (**a**–**c**) or one-way ANOVA (**e**, **f**).

Given that the inhibition of endosomal acidification is a potential strategy for the treatment of SARS-CoV-2 infection, CQ, a clinically used weak base that is able to neutralize proton and thus increase the pH value^8,29^, is in numerous trials to treat SARS-CoV-2-infected patients. However, the treatment outcome is ambiguous^29–31^. Our previous study has demonstrated that macrophages can be polarized by CQ towards an M1 phenotype^8^, prompting us to hypothesize that CQ might strengthen the SARS-CoV-2 infection of AMs through its effect on M1 polarization. Indeed, we found that CQ treatment increased the lysosomal pH of AMs (Fig. 5a and Supplementary Fig. S5a). Unexpectedly, CQ treatment resulted in a decrease in endosomal pH (Fig. 5b). In line with this, we found that CQ treatment increased SARS-CoV-2 replication and load in AMs (Fig. 5c and Supplementary Fig. S5b). To validate these results in vivo, we infected human ACE2 transgenic mice with SARS-CoV-2, following a 5-day CQ treatment (Fig. 5d). RNAscope analysis of the lung tissues showed that CQ treatment markedly increased the virus load (Fig. 5e). Immunostaining showed an increased viral NP level in the CQ-treated lung tissues (Supplementary Fig. S5c). Consistently, aggravating pathological damage was observed (Fig. 5f). Together, these results suggest that CQ may promote SARS-CoV-2 infection in AMs by inducing endosomal acidification.

### M1 AMs facilitate the early infection of the lungs by SARS-CoV-2

**Fig. 6:**
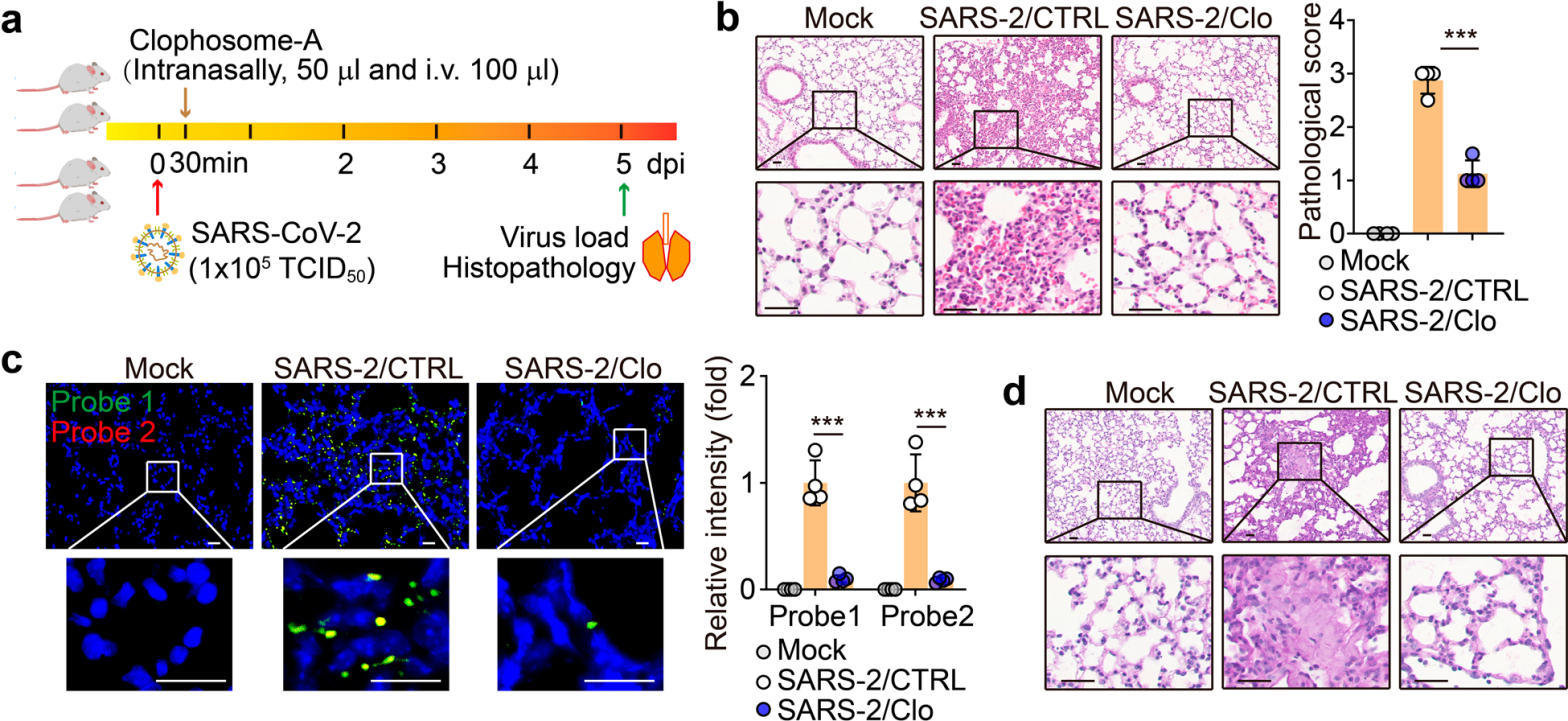
Macrophages depletion attenuated lung damage induced by SARS-CoV-2 in vivo. **a**–**d** hACE2 transgenic mice were infected with SARS-CoV-2 for 30 min, and then administered with a control liposome (CTRL) or clophosome-A (intranasally, 50 μL and i.v. 100 μL) once. The schematic diagram of the experimental design was shown (**a**). The lung tissues were performed the H&E staining (**b**), RNAscope analysis with probe 1 (green color, targeting the positive-sense sequence) and 2 (red color, targeting the negative-sense sequence) (**c**) or PAS staining (**d**). Three lung sections from the left lobe were evaluated for each mouse. The representative image reflected the distributions of damaged lung tissues. Scale bar, 20 μm for **c**, 50 μm for **b**, **d**. The data represent mean ± SD. *n* = 4. ***P < 0.001, by one-way ANOVA (**b**, **c**).

The above data indicated that SARS-CoV-2 seemed to hijack M1 AMs for viral spread, prompting us to investigate the role of AMs in SARS-CoV-2 infection in vivo. Macrophages in the alveoli are highly plastic and can be found under a different polarization state dependent on the local niche^32^. Despite this, the viral pathogen-associated molecular pattern may induce the polarization of AMs toward M1. In vitro treatment of AMs with SARS-CoV-2 indeed resulted in an M1 phenotype with the release of pro-inflammatory cytokines such as IFN-γ, IL-6, and TNF-α (Supplementary Fig. S6a, b). AMs can be effectively depleted by the administration of clodronate^8,33^. Following SARS-CoV-2 infection, we depleted the AMs of the infected hACE2 mice for two days (Fig. 6a) and found that the depletion of AMs mitigated the pathological damage of the lungs, as evidenced by H&E staining (Fig. 6b). The RNAscope analysis also showed that AM depletion led to a dramatic decrease of viral load in the lungs (Fig. 6c). In addition, the real-time PCR and immunostaining of viral NP showed consistent results (Supplementary Fig. S6c, d). Our previous study has shown that SARS-CoV-2 infection causes the production of mucus in the alveoli, thus aggravating the pathological damage^34^. Here, we found that the depletion of AMs relieved this mucous production (Fig. 6d and Supplementary Fig. S6e). Together, these results suggest that AMs play an important role in SARS-CoV-2 infection and pathogenesis.

## Discussion

The pathogenesis of SARS-CoV-2 infection relies on invading the respiratory tract. Given that bronchial and bronchiolar epithelial cells have cilia which can discharge particle-trapping mucus mainly produced by goblet cells, a virus invading the bronchial or bronchiolar epithelium readily causes cough with sputum^35^. In contrast, the alveoli neither have cilia nor produce mucus physiologically. Based on this, a dry cough is a typical symptom for SARS-CoV-2-infected patients^3^, as the virus is likely to mainly invade the alveoli rather than bronchi or bronchioles. Therefore, alveolar macrophages become the first line of defense to initially contact and recognize SARS-CoV-2. In this study, we provide evidence that SARS-CoV-2 hijacks AMs for its own replication and viral spread, leading to its early pathogenesis in the lungs.

Despite tremendous efforts, understanding the role of macrophages in viral infection is largely ambiguous. M1 macrophages have been shown to exert antiviral immunity in certain types of a viral infections such as HIV and SIV^36^. However, in this study, we find that M1 AMs mediate SARS-CoV-2 replication and spread. Intriguingly, M2 AMs show a very weak ability to mediate this process. To explain such a difference, one reason we identified is the mechanical softness of M1 AMs. Physiologically, myosin binds to F-actin and drives the contraction of actin filaments by the released energy from its hydrolysis of ATP^37^. Such tensile force in actin filaments in turn stiffens the F-actin lattice. Thus, the cell stiffness is the collective result of actin polymerization and myosin II-mediated contractile activation^24^. Cell stiffness prevents cellular deformation; however, the softness, in this case, allows M1 AMs to more easily take up SARS-CoV-2 via their deformation. In this study, the signaling pathway triggered to confer M1 AMs a soft phenotype is not elucidated. It is possible that M1 macrophage inducer (IFN-γ or LPS) transduces signals to the cytoskeleton, regulating a soft phenotype, which is worthy of further investigation.

In addition to cellular softness, another explanation for the difference in AMs lies in the altered pH value of the endo-lysosomal system. We find that endosomes are more acidic in M1 AMs than their M2 counterparts; whereas, in contrast, the lysosomes are more alkaline in M1 AMs. Thus, the pH value seems to be inverted in the macrophages’ endosomes and lysosomes regardless of M1 or M2. However, such a pH value pattern is favorable for SARS-CoV-2 survival in M1 rather than M2 AMs. This is because a low endosomal pH favors the virus fusing with the endosomal membrane, thus facilitating the release of the viral genomic RNA to the cytosol, and a high lysosomal pH impairs the degradation of the virus in the lysosomes. Based on these results, an intriguing phenomenon is that endosomal pH and lysosomal pH have an inverse relationship. We did not explore the underlying mechanism in this study; however, one possibility is that endosomes and lysosomes compete for the cytosolic protons using a vacuolar-type H^+^ ATPase pump. In addition, how signals for differential polarization regulate different endo-lysosomal pH levels needs to be addressed in future studies.

It is known that AMs clear billions of inhaled particles, allergens, and microbes daily^38^. To maintain the homeostasis of the alveoli, macrophages can polarize toward M1 or M2 phenotype, thus balancing immune response to anti-infection and inflammation^39,40^. Analysis of 72 individual donors with healthy lungs by flow cytometry has identified that CD206 is commonly expressed by AMs^40^. Given that CD206 is a typical M2 marker, human AMs are seemingly biased to M2-like phenotype, which is not favorable for the spread of invading viruses. This might be a possible explanation for the high percentage of asymptomatic SARS-CoV-2-infected patients. Despite the M2-like polarization, AMs like other tissue macrophages are of high plasticity and can be re-polarized toward the M1 phenotype. Of note, viral RNA or DNA components can act as main PAMPs, which are ready to induce an M1 phenotype of macrophages^41,42^. In this study, we found that SARS-CoV-2 can activate AMs toward the M1 phenotype with the release of proinflammatory cytokines. These cytokines including TNF-α may favor the polarization of macrophages toward an M1-like state^43,44^. Thus, if the viral load reaches a certain level in the alveoli, SARS-CoV-2 might directly reset AMs toward an M1 phenotype and the released proinflammatory cytokines probably further favor M1 AM polarization, thus facilitating viral spread via AMs.

In summary, the data in this study clearly show that alveolar macrophages, by virtue of their polarization state toward either an M1 or M2 phenotype, generate different consequences following SARS-CoV-2 infection. M1 AMs are hijacked by SARS-CoV-2 allowing for viral infection and spread; however, M2 AMs possess the ability to degrade the virus and limit its spread. These findings represent only a beginning in the analysis of the complex interaction of alveolar macrophages with SARS-CoV-2, which may lead to the discovery of new therapeutic targets against this viral infection.

## Materials and methods

### Animals and cell lines

Female ICR, hACE2 mice, 6–8 weeks, were purchased from the Center of Medical Experimental Animals of the Chinese Academy of Medical Science (Beijing, China). These animals were maintained in the Animal Facilities of the Chinese Academy of Medical Science under specific pathogen-free conditions. Animals studies involving SARS-CoV-2 strain WH-09 were performed in an animal biosafety level 3 (BASL3) facility using HEPA-filtered isolators and the procedures were approved by the Institutional Animal Care and Use Committee (IACUC; Protocol Number: ZH20005) of the Institute of Laboratory Animal Science, Peking Union Medical College (BLL20001). Murine studies without viral infection were approved by the Animal Care and Use Committee of the Chinese Academy of Medical Science. Murine macrophage cell line Raw264.7, human monocyte cell line THP-1, and monkey kidney cell line-Vero E6 cells were purchased from the China Center for Type Culture Collection (Beijing, China) and cultured in DMEM or RPMI-1640 medium (Gibco, USA) with 10% FBS.

### Reagents

Chloroquine, Z-VAD-FMK, Jasplakinolide, Lipopolysaccharides, and Cytochalasin D were purchased from Sigma-Aldrich (MO, USA). Recombinant murine IL-4, IFN-γ, and recombinant human IL-4, IFN-γ were purchased from PeproTech (NJ, USA). Clodronate Liposomes were purchased from FormuMax (CA, USA). Latrunculin A was purchased from Millipore (MA, USA).

### Isolation of primary alveolar macrophages and alveolar epithelial type II cells

Primary alveolar macrophages were isolated from murine bronchoalveolar lavage fluid (BALF). Briefly, the mice were anesthetized immediately prior to lavage and the trachea was dissected. Lungs were lavaged five times with 1 mL of PBS and the retained BALF was centrifuged at 600× *g* for 5 min at 4 °C. The pellet harvested was resuspended in RPMI 1640 complete medium and incubated on a culture plate for 2 h. After 2 h, nonadherent cells were removed by gentle washing with PBS. Primary alveolar epithelial (AT2) cells were isolated from hACE2 mice as previously reported^45^. Briefly, mice were perfused with 10 mL cold PBS through the right ventricle. Lungs were filled with 2 mL dispase (BD Bioscience, USA) and low gelling temperature agarose (Sigma Aldrich, USA) before lung tissues were incubated with 2 mL dispase in 37 °C for 20 min. Then, lung tissues were rubbed and the slurry was filtered through 70-μm and 40-μm nylon meshes (JETBIOFIL, China). The cellular suspension was incubated with biotinylated anti-CD45 (Biolegend, clone 30-F11, Cat. 103104), anti-CD16/32 (BD Pharmingen™, clone 2.4G2, Cat. 553143), anti-CD31 (Biolegend, clone MEC13.3, Cat. 102504), anti-TER119 (Biolegend, clone TER119, Cat. 116104) and anti-CD104 (Biolegend, clone 346–11A, Cat. 12603) antibodies at 4 °C for 30 min and then Dynabeads® MyOne^TM^ streptavidin T1 magnetic beads (Thermo Fisher Scientific, Cat. 65601) was added to the cell suspension to exclude leukocytes, monocytes/macrophages, NK cells, neutrophils, endothelial cells, and erythroid cells. A negative selection of fibroblasts was performed by adherence on non-coated plastic plates. Cell purity was assessed routinely by flow cytometry.

### Immunofluorescence

Cells were fixed in 4% paraformaldehyde and permeabilized with 0.2% Triton X-100. Fixed cells were blocked in 5% BSA and incubated with an anti-Lamp2 (Abcam, Cat. ab25339, 1:200) antibody; anti-SARS nucleocapsid protein (Abcam, Cat. Ab273434, 1:200), anti-Rab7 (Abcam, Cat. ab137029, 1:200) antibody or anti-Rab5 antibody (Abcam, Cat. ab18211, 1:200) at 4 °C overnight, cells were washed and incubated with secondary antibodies for 1 h at room temperature. Finally, the slides were counterstained with DAPI and mounted for confocal analysis. The intensity of immunofluorescence was analyzed by Image J 9.0 software.

### Histological and immunohistochemical staining

The lung tissues from mice were fixed in 10% formalin, embedded in paraffin, and sectioned for H&E staining. According to morphological changes after SARS-CoV-2 infection, the lung tissues were graded as mild (1), moderate (2), severe (3), or life-threatening (4). An expert in pathology who was blinded to the experiment gave a score based on the inflammatory cell infiltration, parenchymal pneumonia, alveolar hemorrhage, and bronchiolar/bronchial luminal or alveolar exudate. Immunohistochemical staining was performed according to a protocol as previously described^46^. In brief, the sections of paraffin-embedded tissues were incubated with anti-F4/80 (Santa Cruz Biotechnology, Cat. SC-52664, 1:500) antibody, anti-mucin 1 (1:200, Abcam, Cat. ab45167), anti-mucin 5a (1:200, Abcam, Cat. ab24071), anti-mucin 5b (1:200, Abcam, Cat. ab77995) or anti-SARS nucleocapsid protein (Abcam, Cat. ab273434, 1:1000) antibody at 4 °C overnight. Afterward, slides were sequentially incubated with two HRP-conjugated secondary antibodies for 1 h at room temperature. The slides were incubated with ANO Reagent PPD520 or PPD570 using the PANO 4-plex IHC Kit (Panovue, China) according to the manufacturer’s instructions, followed by counterstaining with DAPI (Thermo, USA) and finally mounting for analysis. The stained lung sections were scanned and digitalized utilizing a TissueFaxs Plus System coupled onto a Zeiss Axio Imager Z2 microscope or Nikon A1 confocal microscope. The intensity of positive staining was analyzed by Image J 9.0 software.

### Real-time PCR

Total RNA was extracted from cells using Trizol (Invitrogen) and was transcribed to cDNA by using a high-capacity cDNA reverse transcription kit (Applied Biosystems, CA). The primer sequences are shown as follows: *GAPDH*, 5’-ACAACTTTGGTATCG TGGAAGG-3’ (sense) and 5’-GCCATCACGCCACAGT TTC-3’ (antisense); *Gapdh*, 5’-AGGTCGGTGTGAACGGATTTG-3’ (sense) and 5’-TGTAGACCATGTAGTTGA GGTCA-3’ (antisense); SARS-CoV-2 primer1 (*ORF1ab*): 5’-CCCTGTGGGTTTTAC ACTTAA-3’ (sense) and 5’-ACGATTGTGCA TCAGCTGA-3’ (antisense); SARS-CoV-2 primer2 (*Nucleoprotein*): 5’-GGGGAACTTCTCCTGCTAGAAT-3’ (sense) and 5’-CAGACATTTTGCTCTCAAGCTG-3’ (antisense); *ACE2*, 5’-CAAGAGCAAACG GTTGAACAC-3’ (sense) and 5’-CCAGAGCCTCTCATTGTAGTCT-3’ (antisense); *Nos2*, 5’-GATGTTGAACTATGTCCTATCTCC-3’ (sense) and 5’-GAACACCACTTT CACCAAGAC-3’ (antisense); *Arg1*, 5’-CAAGACAGGGCTCCTTTCAG-3’ (sense) and 5’-TGGCTTATGGTTACCCTCCC-3’ (antisense). Real-time PCR was performed using ABI QuantStudio 3 (Applied Biosystems, CA, USA). Values are means ± SD from three independent experiments which were performed in duplicate. Statistical comparisons among groups were performed using a Student’s *t*-test. Values of all parameters were considered statistically significant at a value of *P* < 0.05.

### RNA in situ hybridization (RNA-ISH)

RNA-ISH was performed on primary alveolar macrophages grown on glass coverslips or paraffin-embedded 5 μm lung tissue sections using the RNAscope Multiplex Fluorescent Assay v2 according to the manufacturer’s instructions (Advanced Cell Diagnostics, USA). Briefly, cells were fixed in 4% paraformaldehyde and incubated with hydrogen peroxide at RT for 10 min and 1:15 diluted Protease III at RT for 10 min. Lung tissue sections were deparaffinized with xylene and rehydrated with graded ethanol, incubated with hydrogen peroxide, and then boiled for 15 min in Target Retrieval buffer, followed by incubation with Protease Plus for 15 min at 40 °C. Slides were hybridized with SARS-CoV-2 probes in a hybridization oven at 40 °C for 2 h, and the fluorescent signals were amplified according to the manufacturer’s protocol. The cells grown on glass coverslips and stained lung sections were scanned and digitalized utilizing a TissueFaxs Plus System coupled onto a Zeiss Axio Imager Z2 microscope. The intensity of fluorescence and positive cell rate were analyzed by Image J 9.0 software.

### Lysosomal pH measurement

LysoSensor^TM^ Yellow/Blue DND-160 (Thermo Fisher, USA) was utilized to quantify the pH of macrophage lysosomes according to the manufacturer’s guidelines, which exhibits the pH-dependent dual-excitation spectra in living cells. LysoSensor^TM^ Yellow/Blue DND-160 has a predominantly yellow fluorescence in acidic environments and a blue fluorescence in an alkaline environment. In brief, cells were treated with 5 μM LysoSensor^TM^ Yellow/Blue DND-160 in the prewarmed medium for 5 min at 37 °C. After washing twice with cold PBS, the labeled cells were incubated at 37 °C for 5 min with 10 μM monensin and 10 μM nigericin in Living Cell Imaging Solution (Thermo Fisher, USA). The fluorescent intensity was measured at Ex-330/Em-550 and Ex-380/Em-550. The standard curve of pH value was performed by Intracellular pH Calibration Buffer Kit (Thermo Fisher, USA). For the relative acidity of the lysosome, macrophages were incubated with 5 μM LysoSensor^TM^ Green DND-189 (Thermo Fisher, USA) for 30 min under appropriate growth conditions. Then the loading solution was replaced with a fresh medium and the stained cells were observed and digitalized utilizing a Nikon A1 confocal microscope. The relative intensity was analyzed by Image J 9.0 software.

### Endosomal acidity detection

For detecting the endosomal acidity, pHrodo^TM^ red dextran (Thermo Fisher, USA) was utilized, which possesses a pH-sensitive fluorescent emission that increases in intensity with increasing acidity and is essentially non-fluorescent in the extracellular environment. Following the manufacturer’s guidelines, AMs or PAECs were cultured with 50 μg/mL pHrodo^TM^ red dextran in Live Cell Imaging Solution for 10 min at 37 °C. After washing with a pre-warmed medium twice, the cells were analyzed by flow cytometry and imaged by a Nikon A1 confocal microscope with an appropriate filter.

### Animal experiments and treatment protocol

hACE2 mice were infected with SARS-CoV-2 (1 × 10^5^ TCID_50_) by intratracheal administration and then treated with vehicle control (H_2_O or control liposome), CQ (35 mg/kg, i.p.) or CQ combined with Clodronate Liposomes (50 μL by intratracheal administration and 100 μL by intravenous injection, only once) once a day for 5 days (*n* = 4 mice/group). After 5 days of treatment, mice were euthanized and lung tissues were collected for real-time PCR assay and histological and immunohistochemical staining.

### Cell viability detection

To assess the cell viability of AMs infected with SARS-CoV-2, the CellTiter-Glo^®^ Luminescent Cell Viability Assay Kit (Promega, USA) was used according to the manufacturer’s instructions. In brief, 1 × 10^5^/well AMs were placed in 24-well plates and infected with 10^5^ TCID_50_ SARS-CoV-2 for 4 h. Cells were then lysed with Dual-Luciferase Reporter Assay System reagent and the luciferase signal was determined by a microplate luminometer.

### Quantification and statistical analysis

All experiments were performed at least three times. Results are expressed as mean ± SD as indicated and analyzed by two-tailed Student’s *t*-test or One-way ANOVA followed by Bonferroni’s test or Kruskal–Wallis test. The *P-*value < 0.05 was considered statistically significant. The analysis was conducted using the Graphpad 8.0 software.

## Supplementary information

Supplementary Information
