# Supplementary material for: Distinct uptake, amplification, and release of SARS-CoV-2 by M1 and M2 alveolar macrophages
Source: Cell Discov. 2021 Apr 13;7:24. doi: 10.1038/s41421-021-00258-1 (PMC8043100; doi:10.1038/s41421-021-00258-1)
Supplement: Supplementary file 1 — Supplementary Information [file 41421_2021_258_MOESM1_ESM.pdf]

Supplementary figure legends

Figure S1

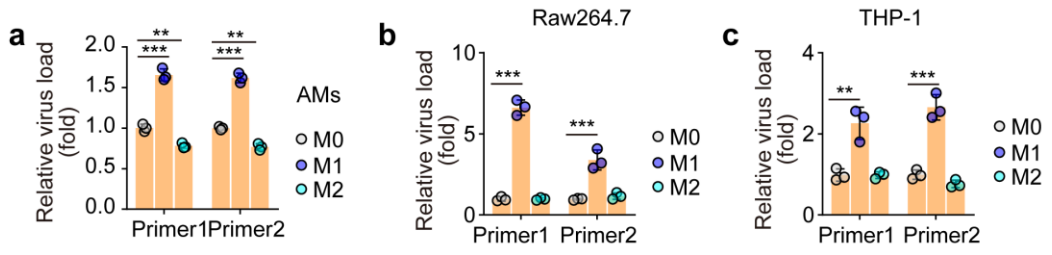

Supplementary Fig. S1 M1 macrophages facilitate SARS-CoV-2 infection. **a, b** Primary alveolar macrophages (AMs) or Raw264.7 were polarized to M1 by IFN-γ (20 ng/ml) and LPS (100 ng/ml) or M2 by IL-4 (20 ng/ml) for 24 hr, and then infected with SARS-CoV-2 at the ratio of 1:1 ($TCID_{50}$: cells) for 2 hr. The viral load was analyzed by real-time PCR with specific primers 1 and 2. **c** The same as (**a**, **b**), except that THP-1 were pretreated with PMA (100 ng/ml) for 48 hr. The data represent mean ± SD of 3 independent experiments. ** $p<0.01$, *** $p<0.001$, by one-way ANOVA (**a**-**c**).

Figure S2

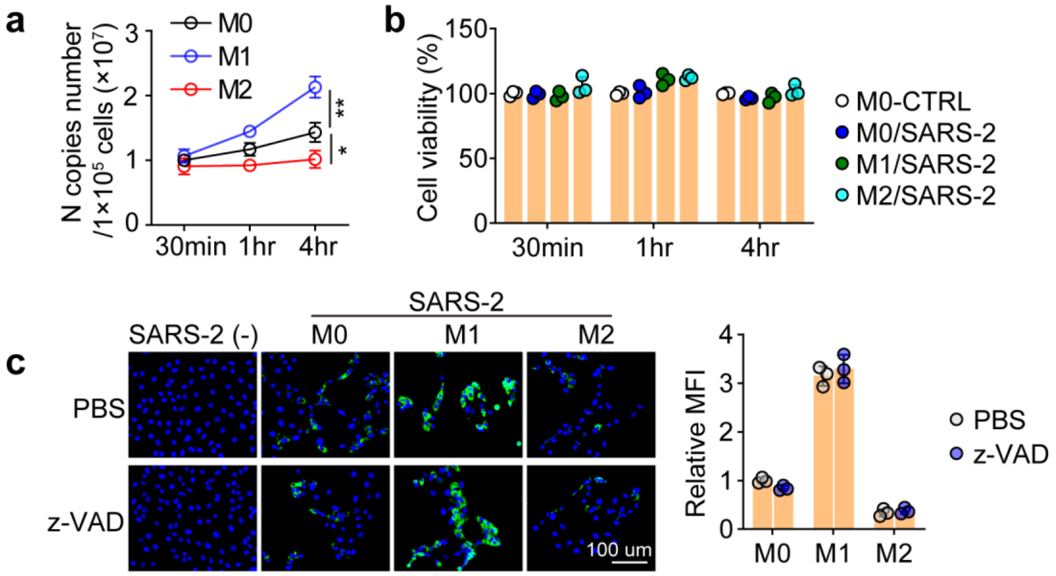

Supplementary Fig. S2 M1 AMs enable SARS-CoV-2 replication. **a**, **b** M0, M1 and M2 AMs were infected with SARS-CoV-2 for 30 min. Then, the viruses were removed and the cells were cultured with virus-free medium for another 30 min, 1 hr or 4 hr. The viral load was analyzed by real-time PCR (**a**). Cell viability was measured by a microplate luminometer (**b**). The M0 AMs without SARS-CoV-2 infection served as a control (M0-CTRL). **c** M0, M1 and M2 AMs pretreated with z-VAD-FMK (50 μM) for 2 hr were infected with SARS-CoV-2 for 4 hr. The supernatants were collected to infect Vero E6 cells for 48 hr. Cells were stained with anti-NP antibody and observed by confocal microscopy (**c**). Scale bar, 100 μm. The data represent mean ± SD of 3 independent experiments. * $p<0.05$, ** $p<0.01$, by one-way ANOVA (**a**).

Figure S3

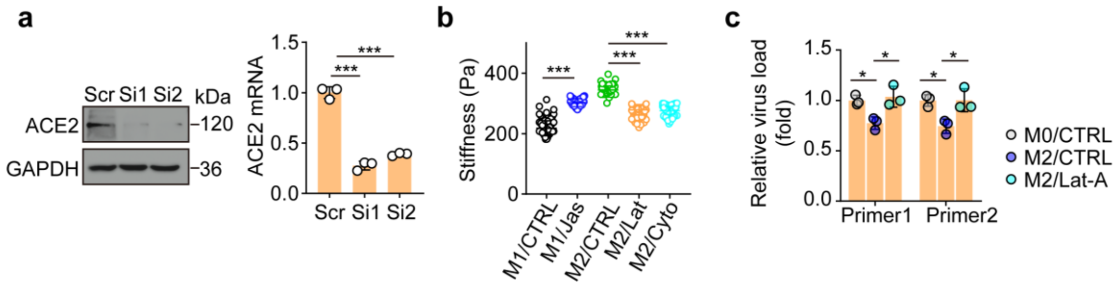

Supplementary Fig. S3 The stiffness of macrophages affected the uptake of viruses. **a** AMs isolated from hACE2 transgenic mice were transfected with scrambled or *ACE2*-siRNAs. The knockdown efficiency of *ACE2* was evaluated by western blotting (left) and real-time PCR (right). **b** M1 or M2 AMs were treated with control vehicle (DMSO), jasplakinolide (Jas, 50 nM) for 12 hr, latrunculin A (Lat, 2 μM) for 12 hr or cytochalasin D (Cyto, 1 μM) for 4 hr. Cellular stiffness was detected by atomic force microscopy (AFM). **c** M2 AMs pretreated with Lat (2 μM) for 12 hr were infected with SARS-CoV-2 for 2 hr. The viral load was analyzed by real-time PCR. The data represent mean ± SD of 3 independent experiments. * $p<0.05$, *** $p<0.001$, by one-way ANOVA (**a**,**c**) or Kruskal-Wallis test (**b**).

Figure S4

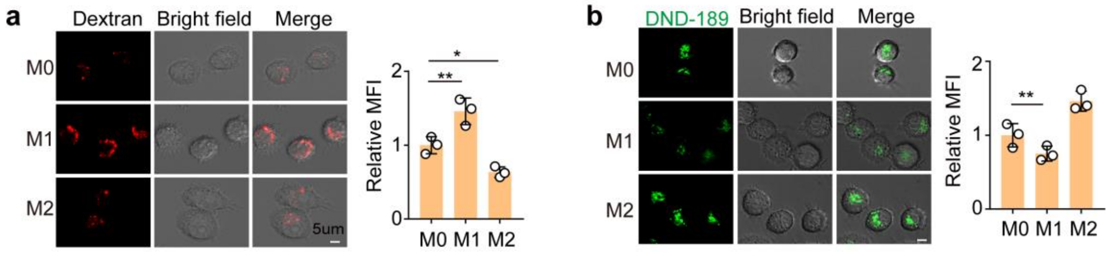

Supplementary Fig. S4 M0, M1 and M2 AMs showed different pH values in endosomes or lysosomes. **a** M0, M1 and M2 AMs were stained with pHrodo™ Red dextran (50 μg/ml) for 10 min at 37°C and observed by a confocal microscope. Scale bar, 5 μm. **b** The same as (**a**), except that cells were stained with LysoSensor™ Green DND-189 (5 μM) for 30 min at 37℃. Scale bar, 5 μm. The data represent mean ± SD of 3 independent experiments. * $p<0.05$, *** $p<0.001$, by one-way ANOVA (**a**, **b**).

Figure S5

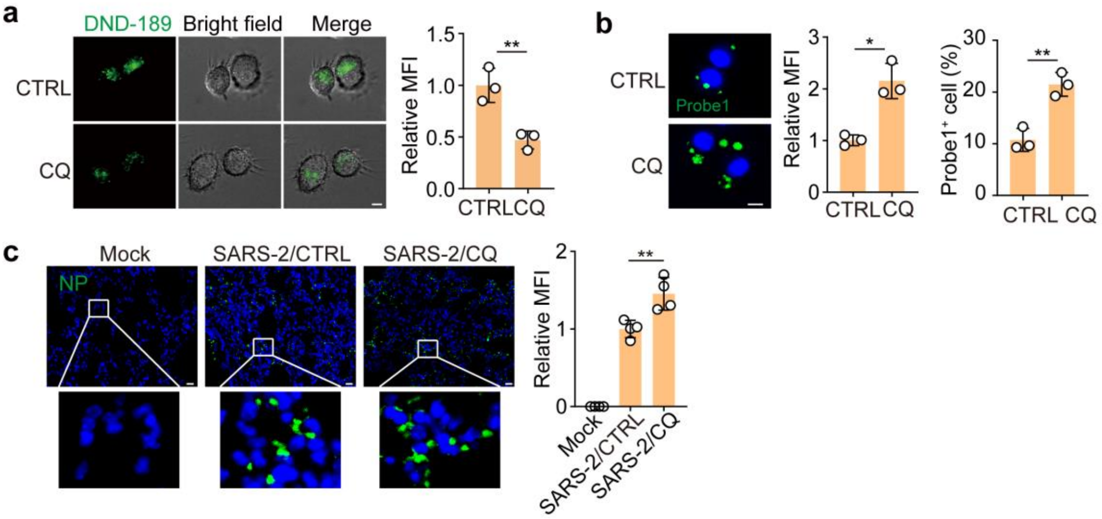

Supplementary Fig. S5 CQ enhanced SARS-CoV-2 infection *in vivo*. a AMs pre-treated with CQ (10 μM) for 24 hr were stained with LysoSensor™ Green DND-189 and observed by confocal microscopy. Scale bar, 5 μm. **b** M0 AMs pretreated with CQ (10 μM) for 24 hr were infected with SARS-CoV-2 for 30min. Cells were fixed for RNAscope with specific Probe 1, which targets the positive-sense sequence. Scale bar, 5μm. **c** hACE2-transgenic mice were infected with SARS-CoV-2, and then treat-ed with or without CQ (35 mg/kg, i.p.) once every day for 5 days. The lung tissues were fixed to perform the immunohistochemical staining of NP (n = 4 mice). The data represent mean ± SD. In **a** and **b**, n = 3 independent experiments. * $p<0.05$, ** $p<0.01$, by two-tailed student's t-test (**a**, **b**) or one-way ANOVA (**c**).

Figure S6

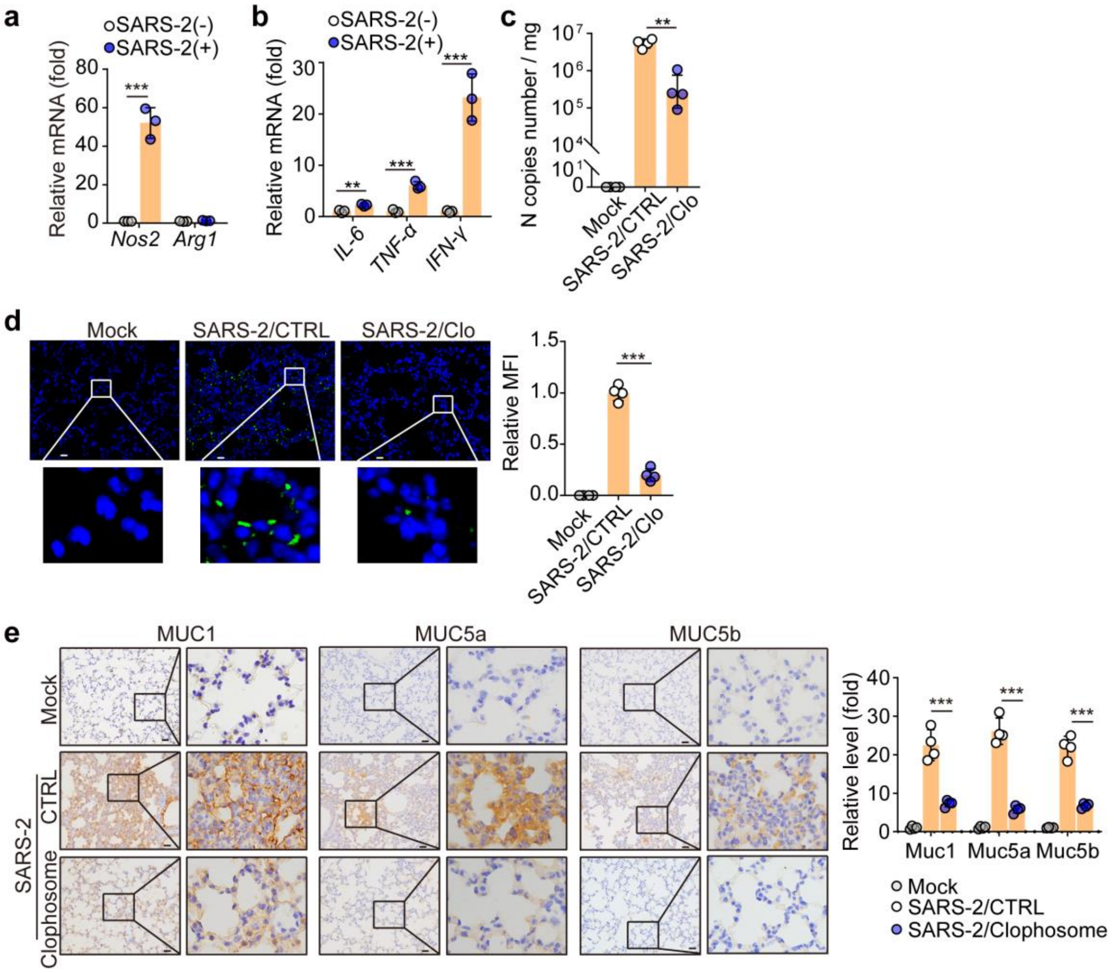

Supplementary Fig. S6 Macrophage depletion reduced the infection of SARS-CoV-2 and the production of mucins *in vivo*. **a-b** M0 AMs were infected with SARS-CoV-2 for 24 hr. The expression of *Nos2*, *Arg1* (**a**), *IL-6*, *TNF-α* or *IFN-γ* (**b**) was determined by real-time PCR. **c-e** hACE2-transgenic mice were infected with SARS-CoV-2, and then treated with a control liposome or clophosome-A (both i.v. 100 μl and nasally 50 μl). The lung tissues were used to perform the real-time PCR (**c**, n=4 mice), the immunohistochemical staining of NP (**d**, n = 4 mice) or mucins 1, 5a and 5b (**e**, n = 4 mice). The data represent mean ± SD. In **a**, **b** n = 3 independent experiments. * p<0.05, ** p<0.01, by two-tailed student's t-test (**a, b**) or one-way ANOVA (**c**, **d, e**).
